# Transcriptional landscape of cellular networks reveal interactions driving the dormancy mechanisms in cancer

**DOI:** 10.1038/s41598-021-94005-x

**Published:** 2021-08-04

**Authors:** Dilara Uzuner, Yunus Akkoç, Nesibe Peker, Pınar Pir, Devrim Gözüaçık, Tunahan Çakır

**Affiliations:** 1 Department of Bioengineering, Gebze Technical University, 41400 Kocaeli, Turkey; 2 Koç University Research Center for Translational Medicine (KUTTAM), Zeytinburnu, 34010 Istanbul, Turkey; 3 Koç University School of Medicine, Sarıyer , 34450 Istanbul, Turkey; 4 SUNUM Nanotechnology Research and Application Center, Tuzla, 34956 Istanbul, Turkey

**Keywords:** Gene regulatory networks, Computational models

## Abstract

Primary cancer cells exert unique capacity to disseminate and nestle in distant organs. Once seeded in secondary sites, cancer cells may enter a dormant state, becoming resistant to current treatment approaches, and they remain silent until they reactivate and cause overt metastases. To illuminate the complex mechanisms of cancer dormancy, 10 transcriptomic datasets from the literature enabling 21 dormancy–cancer comparisons were mapped on protein–protein interaction networks and gene-regulatory networks to extract subnetworks that are enriched in significantly deregulated genes. The genes appearing in the subnetworks and significantly upregulated in dormancy with respect to proliferative state were scored and filtered across all comparisons, leading to a dormancy–interaction network for the first time in the literature, which includes 139 genes and 1974 interactions. The dormancy interaction network will contribute to the elucidation of cellular mechanisms orchestrating cancer dormancy, paving the way for improvements in the diagnosis and treatment of metastatic cancer.

## Introduction

Cancer is one of the most fatal and refractory diseases. Although advances in medicine and technological developments now allow treatment of many types of cancer, especially if patients were diagnosed at earlier stages; diagnosis of advanced disease, metastatic relapse and treatment at these late stages still remains as a difficult challenge. There is compelling evidence that dormant cancer cells are responsible for cancer recurrence^1^. Some of the disseminated tumor cells might escape immune surveillance and develop resistance against cancer treatment modalities. These single cells or micrometastases formed by disseminated tumor cells are beyond the detection limit of current cancer diagnosis techniques, and untraceable through CT scans, MRI approaches and PET-scans. Thus, residual disseminated tumor cells remain in the body, enter a dormant state (hereafter called dormancy) and cause recurrence of the disease months or even years after "the cure" (5 years tumor-free survival).

Cancer cell dormancy is a complex cellular phenomenon and remains to be explored. However, recent studies suggested that there are two major mechanisms of cancer dormancy: Tumor mass dormancy and cellular dormancy^2^. Tumor mass dormancy refers to the stagnation in tumor mass growth, when cell division and cell death take place at the same rate, keeping the total number of cells constant. Cell numbers in tumors, hence the tumor mass, depend on vascular supply of blood and anti-tumor immune responses. In cellular dormancy however, cell-intrinsic mechanisms that control proliferation and cell cycle entry are dominant. Rather than cell division versus death rates of cancer cells, in cellular dormancy, intrinsic mechanisms leading to a quiescence-like stage and cell cycle arrest are in play. Experimental models indicate that cellular dormancy can be triggered by drugs, growth factor/hormone deprivation, hypoxia, tumor microenvironment components such as extracellular matrix signals and stromal cell interactions^1^. An important characteristic of dormant cells is their ability to exit the dormancy state and continue to proliferate. Little is known about reasons and mechanisms of entry to dormant state and factors that trigger cell reactivation. Recent studies employed transcriptome-based analyses of dormant cells and revealed potential target genes that may play a role in dormancy in different cancer types^3,4^.

Network-based computational methods are frequently used in the systems biology field to elucidate disease-associated molecular mechanisms from omics data. Integration of omics data with biological networks is a promising approach for better understanding the disease etiology and to unravel efficient biomarkers and drug targets^5^. In the literature, numerous techniques were proposed for integrating omics data with biological networks. One of these techniques is active-module detection through network projection of omics data^6^, known also as subnetwork discovery. KeyPathwayMiner^7^ and BioNet^8^ are two powerful subnetwork discovery tools that are used for data integration^9^. KeyPathwayMiner has been applied to data from several diseases, including ovarian cancer^10^, breast cancer^11^ and multiple sclerosis^12^. Similarly, BioNet has been applied to data from prostate cancer^13^, breast cancer^14^, and hepatocellular carcinoma^15^, among others.

Although in the last couple of years, there is a sharp increase in the number of studies generating transcriptomic data from dormant cancer cells, a meta-analysis of those datasets is still missing. Moreover, available studies mostly lack a network-based advanced analysis of the gene expression signatures of dormant states. Here, by mapping 21 comparisons from 10 transcriptome studies on human or mouse protein–protein interaction (PPI) networks and gene-regulatory (GR) networks, we provide a comprehensive transcriptome-based catalogue of dormancy-related genes and interactions.

## Results and discussion

### Discovery of PPI and GR subnetworks from dormancy transcriptome datasets

The 10 dormancy-associated transcriptome datasets were obtained from GEO database (Supplementary Table S1). 7 datasets were collected from human cell lines and the other three were collected from mouse cell lines. Datasets were obtained using either microarray, RNAseq or single-cell RNAseq methods. The 10 datasets included 7 different cancer types (bladder cancer, acute lymphoblastic leukemia, colorectal cancer, acute myeloid leukemia, breast cancer, prostate cancer, and myeloma cells).

KeyPathwayMiner (KPM) and BioNet were applied separately to each dormancy–cancer comparison to extract subnetworks that are enriched in significantly changed genes. As a result, we obtained 34 subnetworks for PPI network-based analyses, and 35 subnetworks for GR and TF-free-GR network-based analyses of 21 comparisons. Sizes of the discovered subnetworks are shown in Supplementary Table ﻿S2. No subnetworks were found by BioNet in PPI and GR subnetwork discovery analyses for 8 and 7 of comparisons respectively. However, when both BioNet and KPM discover subnetworks, BioNet usually reports a bigger subnetwork. Besides, majority of the genes in the dormancy-interaction network have appeared in subnetworks identified either by both tools or only by BioNet (Supplementary Fig. S1).

Thereafter, we performed enrichment analysis for each subnetwork to show whether they were enriched with genes associated with dormancy. Even though the subnetworks were based on data from different cancer types and different cell lines, genes in the subnetworks were mainly enriched in terms that are known to be associated with dormancy-related mechanisms such as extracellular matrix organization, response to stress and cell cycle regardless of the network type they were derived from (results not shown). Indeed, during cellular dormancy, tumor cells repress cell cycle driving pathways and activate cell cycle inhibitory pathways in order to halt proliferation^1^. For example, dormant cancer cells directly interact with extracellular matrix, and cell–matrix contact regulates tumor cell growth, migration, differentiation and survival^2^. Stress conditions such as hypoxia, nutrient deprivation, and chemotherapy induce dormancy, hence activation of stress response pathways during dormancy contribute to the resistance of these cells to these unfavorable conditions^16^. Convergence of enrichment analysis results to above mentioned pathways justified our computational approach for the discovery of key genes in dormancy mechanisms using genome-wide networks.

### Integrated PPI and GR subnetworks pinpoint genes playing roles in dormancy associated molecular mechanisms

In the next step, KPM and BioNet results of each dormancy–cancer comparison were combined to eliminate algorithm-specific effects on the results. Due to limited number of datasets in literature that compares cancer and dormancy data, datasets utilized in this study were obtained from different experimental conditions, cancer types and/or organisms. By consolidating the results of two different algorithms and scoring genes based on the number of appearances in all comparisons, we minimize the effect of these differences. We integrated all subnetworks to score genes based on their appearance in subnetworks. We aimed to extract the most important genes across subnetworks. Score tables were generated for PPI network and GR network analyses separately.

The score table of PPI subnetworks contains 4459 genes that appeared at least in one subnetwork. The genes were filtered based on their appearance in multiple dormancy–cancer comparisons or datasets and based on their significant upregulation (see “Methods” section for details). The filtered score table includes 74 genes based on significance score filter (Supplementary Table ﻿S3). The score tables of GR and TF-free GR subnetworks contain 2196 and 2969 genes respectively and filtered score tables include 70 and 111 genes (Supplementary Table ﻿S3). The genes in filtered score tables also have interactions with each other in the associated genome-wide networks. 47 out of 74 genes interact via 67 edges in PPI network, 15 out of 70 genes interact via 16 edges in GR network and 110 out of 111 genes interact with 1892 edges in TF-free GR network (Supplementary Fig. ﻿S2).

The top 10 genes in filtered score tables in both PPI and GR subnetworks were mainly associated with cell cycle and extracellular matrix (Table 1). Details on these genes in terms of the number of comparisons they were identified in and the corresponding tumor type (liquid or solid) are given in Supplementary Table S4. In concordance with the literature, our results demonstrated that ECM organization and cell cycle control are key pathways in dormancy mechanisms in different cancer types^2,3^. Based on this, genes that are important according to our study were examined in detail to discover other unknown pathways having role in dormancy mechanisms. Clusterin (*CLU*), H2B clustered histone 21 (*HIST2H2BE*) and F-box protein 32 (*FBXO32*) were found among top 10 genes of all PPI, GR and TF-free GR filtered lists. CLU reduces the sensitivity of prostate cancer cells to chemotherapy and it is highly expressed in drug-resistant cancer cells^17^ In addition, the CLU protein has been shown to induce epithelial–mesenchymal transition (EMT) in lung cancer^18^. Nayak et al. showed that HIST1H2BE is overexpressed in treatment resistant breast cancer cells^19^. FBXO32 is ubiquitinated to stabilize CtBP1, which induces transcription of genes for creating suitable microenvironment for EMT progression^20^. CDKN2B/p15INK4B was reported to attenuate the growth capacity of the tumors and sustain dormancy phenotype. CDKN2B binds to CDK4/CDK6, and controls transition of proliferative state and it also inhibits ENO1, a glycolytic enzyme, whose activation required for aerobic glycolysis^21^.

**Table 1:** Top 10 genes in the filtered score lists.

PPI		GR		TF-free GR	
Gene symbol	Significant Score	Gene symbol	Significant Score	Gene symbol	Significant Score
*CLU*	9	*CLU*	7	*CLU*	8
*APP*	7	*CDKN2B*	6	*NEU1*	7
*HIST1H1C*	6	*HIST2H2BE*	6	*OPTN*	6
*HIST2H2BE*	6	*FBXO32*	6	*HSPA1B*	6
*OPTN*	6	*CTSB*	6	*NR1D1*	6
*FBXO32*	6	*THBS1*	5	*CDKN2B*	6
*PLAUR*	5	*VEGFA*	5	*HIST2H2BE*	6
*CTSB*	5	*PLK2*	5	*FBXO32*	6
*THBS1*	5	*BMF*	5	*CTSB*	6
*CDKN2B*	5	*EPAS1*	5	*ABCG1*	6

Many potential top hits from Table 1 have not been studied in cancer dormancy context before, yet several studies revealed their function in metastatic outgrowth, escape from immune surveillance, resistance to cell death and stemness. OPTN was identified as a key regulator of cell cycle arrest and stemness in cancer cells^22^. Ectopic expression of NEU1 suppressed migration and invasion in in vitro cancer models. Also, administration of NEU1 expressing cells into mice prevented metastasis significantly. On the other hand, loss of NEU1 was associated with increased mobility and invasiveness^23,24^. Also, targeting of NEU1 was reported to attenuate drug resistance^25^. In mice, loss of the cholesterol transporter *ABCG1* resulted in excessive lipid accumulation in macrophages with an elevated proinflammatory cytokines secretion, leading to altered tumor-killing capacity of the macrophages^26^. Increased expression of *ABCG1* was documented in 3D tumoroids and significantly correlated with low Ki67 levels^27^.

In a﻿ddition to transcriptome data, one of the effective factors in this study is interactome data. Interactome databases store experimentally validated interactions or interactions predicted by methods such as text-mining. Only experimentally validated interactions were used in this study. However, the information contained in these databases does not yet include all protein–protein interactions due to the technical difficulties in detecting the interactions. Therefore, there is no interaction information for a set of genes in the analyzed transcriptome datasets, and these genes were automatically excluded from our subnetwork-based analysis approach. In order to dissect their possible roles in dormancy, a separate analysis was performed for the genes in the transcriptome datasets with no associated interactome data. These genes were scored based on the number of transcriptome datasets in which they were significantly changed (Supplementary Table S5﻿). The highest scoring genes in the analysis are not associated with autophagy, ECM, cell cycle, or dormancy. This shows that the absence of these genes in our subnetwork analysis does not cause a loss of information. On the other hand, their significance in multiple dormancy-related datasets implies that, although experimentally not reported, they may have potential roles in dormancy mechanisms.

### Network based meta-analysis of transcriptome datasets leads to a dormancy–cancer interaction network

**Figure 1:**
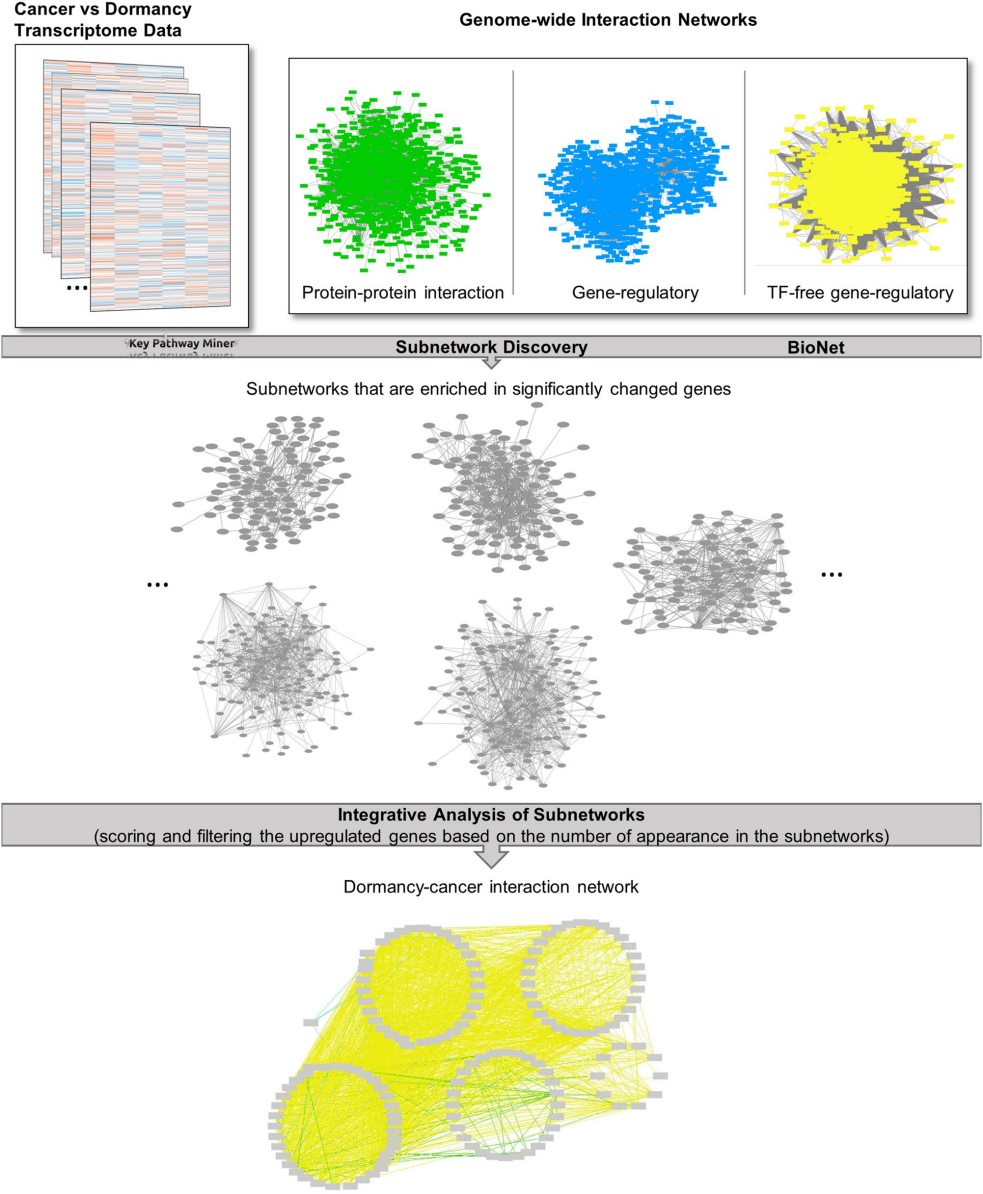
Summary of computational approach to construct the dormancy interaction network. Firstly, cancer–dormancy transcriptome data were downloaded from GEO database and Genome-wide interaction networks were obtained. Then, KPM and BioNet, two subnetwork discovery tools, were performed and subnetworks that are enriched in significantly changed genes were identified. Finally, the upregulated genes were scored and filtered based on the number of appearances in the subnetworks and according to this filtering, the dormancy-interaction network was constructed (see also “Methods”).

Finally, we created a “consensus dormancy-interaction network” composed of the union of the genes in the three filtered lists and their interactions. Since down-regulated genes were mainly associated with cell cycle activation, the constructed dormancy–interaction network focused mainly on up-regulated genes during dormancy. These up-regulated genes have a high potential to be associated with molecular mechanisms of dormancy activation. Indeed, many of the genes in dormancy–interaction network were associated with cancer or dormancy pathways. Figure 1 summarizes our computational approach to construct the dormancy interaction network from transcriptome data and molecular interaction networks.

**Figure 2:**
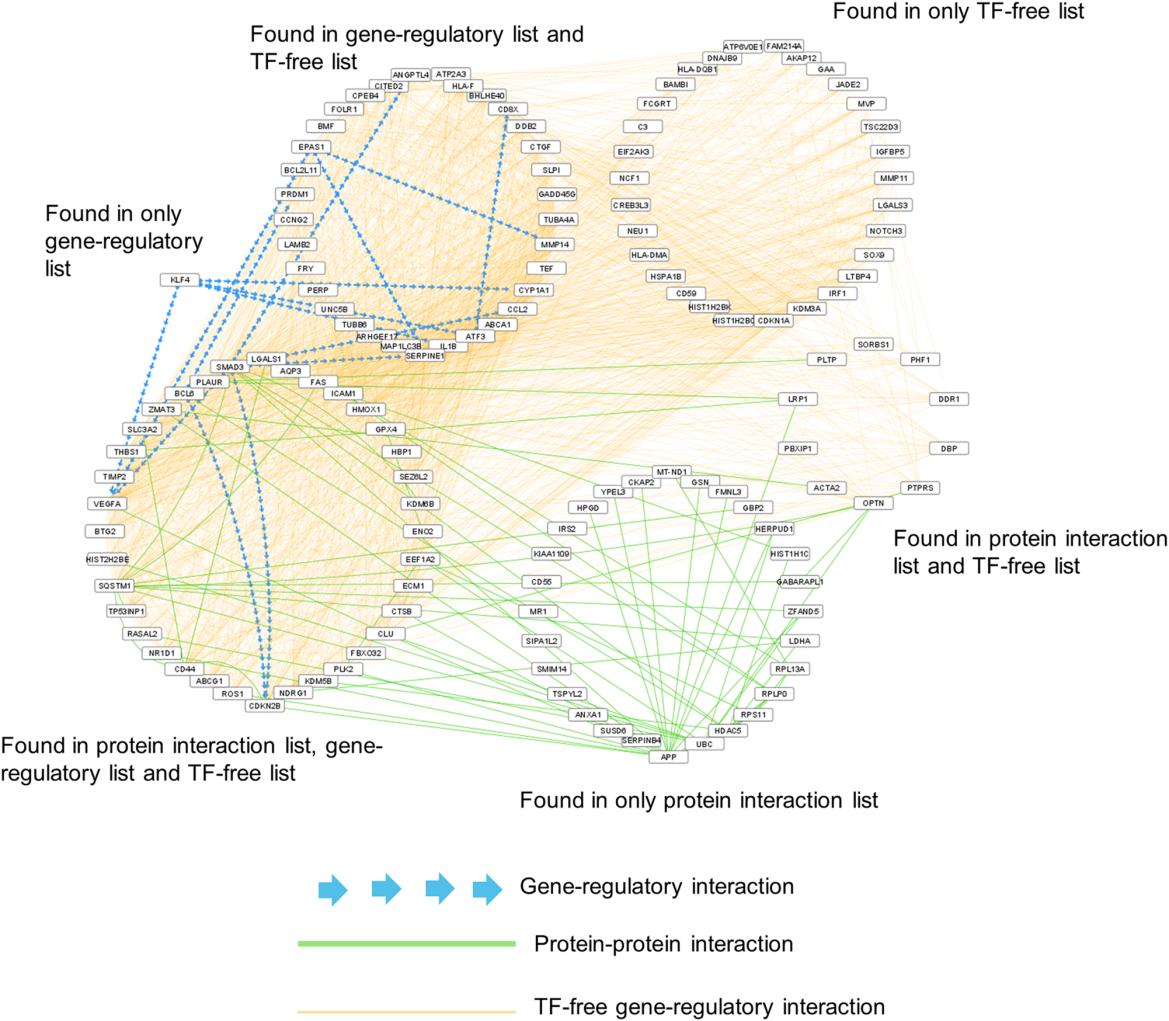
Dormancy-interaction network. The network was constructed by integrative analysis of all subnetworks. Dormancy-interaction network includes genes found in different subnetwork analyses (shown in different clusters). Gene regulatory interactions between genes are shown by blue arrows, the direction of the arrow represents the direction of the interaction. Protein–protein interactions are shown by green lines and TF-free gene-regulatory interactions by orange lines.

The dormancy-interaction network included 139 genes and 1974 interactions (Fig. 2). Enrichment analysis of these 139 genes led to terms that pinpoint cancer cell dormancy mechanisms, such as “negative regulation of programmed cell death”, “cell communication” and “response to stress” (Supplementary Table S6﻿). The network included 36 genes that appeared in the score lists of all three network types. Enrichment analysis of the intersection-genes revealed that they were mainly associated with “cell-death” and “cell-cycle arrest”, similar to union of networks (Supplementary Table S6﻿). Most of these genes were reported to be related with several types of cancer in literature such as *EPAS1*^28^, *SMAD3*^29^, *NOTCH3*^30^. ENO2 has a role in adaptation to serum starvation, hypoxia and chemotherapy in glioblastoma^31^. BTG2 and CDKN2B cause cell cycle arrest in different cancers and BTG2 is upregulated in quiescent cells^32^ TIMP-2 also causes cell cycle arrest and inhibits angiogenesis by de-phosphorylation of VEGF^33^. HO-1 and VEGF-A induce angiogenesis. VEGF-A is found to be upregulated in our study. Although its inhibition has been associated with dormancy^34^, VEGF-A plays a role as not only angiogenesis induced factor but also tumor-induced immunosuppressor, which can explain its function in dormant cells^35^. On the other hand, VEGF-A has the highest degree (96 interactions) in the dormancy–interaction network, and the 3 cancer-related transcription factors (EPAS1, KLF4 and SMAD3) that control VEGF-A also appear in the dormancy interaction network. The fact that VEGF-A has a high number of interactions in the predicted dormancy network and the increase in the expression levels of transcription factors that are effective in VEGF-A regulation supports that VEGF-A plays an important role in the dormancy mechanism. THBS1 is another angiogenesis regulator, and its upregulation was associated with dormancy^4,36^. Another high degree gene in the network is the cyclin dependent kinase inhibitor CDKN1A (93 interactions), which was reported to arrest cells in the G0/G1 phase. Also, increased expression of CDKN1A was previously associated with dormancy^37^. All interactions of CDKN1A in the dormancy-interaction networks are TF-free regulatory interactions, which further supports our choice of including the TF-free network in our approach. There are many interactions in the dormancy interaction network originating from the TF-free network. This suggests that the genes associated with dormancy may not physically interact, but rather regulated by similar transcription factors. Most of the genes that were commonly found in all three network types were not directly linked with dormancy previously, but the genes regulate critical dormancy pathways including hypoxia, extracellular matrix organization and angiogenesis^2^. 15 out of 139 dormancy interaction network genes were previously associated with dormancy (Table 2).

**Table 2:** The genes in the dormancy–interaction network and the mechanisms associated with dormancy.

Gene symbol	Role in dormancy	Model	References
*AKAP12*	Inhibits proliferation and migration of colorectal cancer cells	In vitro LoVo colorectal cancer cell and in vivo mouse xenografts	^58^
*CD44*	Expressed in dormant breast cancer cells and hepatocellular carcinoma cells	In vitro MCF-7 breast cancer cell line treated with the farnesyl transferase inhibitor (FTI) In vitro HepG2 hepatocellular cancer cell line. Dormancy condition promoted with matrix stiffness	^59,60^
*CDKN1A*	Induces dormancy and G1 cell cycle arrest	In vitro human breast epithelial MCF10A cell line	^37^
*DDR1*	Dormancy signature in breast cancer	Microarray data of 51 breast cancer cell lines	^61^
*EPAS1*	Induces dormancy in lung cancer. Helps adaptation of cancer cells to hypoxic environment	Ex vivo culture of lung cancer tissue-originated spheroids in Matrigel growth factor reduced matrix	^28^
*GSN*	Induction causes G2/M cell cycle arrest of tumor cells	In vitro KU-7 and UMUC-2 bladder cancer cell lines. In vivo KU-7 cell xenografts	^62^
*HBP1*	Regulates dormancy in breast cancer	3D in vitro model (GELFOAM™). MDA-MB-231 breast cancer cells	^63^
*IL1B*	Activates dormant CD34^+^/CD38^−^ acute myelogenous leukemia cells	In vitro EOL‐1R cell line	^64^
*NDRG1*	Promotes dormancy in prostate cancer. Suppresses metastasis	PC3 mm prostate cancer cell line, in the presence of the conditioned medium of human bone marrow stromal cells	^65^
*NOTCH3*	Reduced levels are detected in dormant tumor of T-cell acute lymphoblastic leukemia	In vivo MOLT-3 cell xenografts	^30^
*PLAUR*	Decreased levels in dormant epidermoid carcinoma cells. Overexpression interrupts dormancy	In vivo human epidermoid carcinoma HEp3 cells passaged on chorioallantoic membranes	^66^
*SMAD3*	Controls transition between dormancy and active states of tumor-propagating cancer cells of squamous cell carcinoma cells	ATAC-seq and ChIP-seq on squamous cell carcinoma cell lines	^29^
*THBS1*	Induces dormancy in breast cancer and glioblastoma	Microarray data of 51 breast cancer cell lines 153 glioblastoma patient samples from TCGA	^4,36^
*TIMP2*	Regulates dormancy in sarcoma. Induces cell cycle arrest and modulates angiogenesis	In vivo myxoid liposarcoma xenografts	^33^
*TSC22D3*	Encodes for GILZ protein. GILZ expression is low in dormant melanoma cells than the maternal cells	In vitro HBL human melanoma cells and in vivo transgenic B16F1 melanoma mice	^67^

It has been known that dormant cancer cells have molecular similarities with quiescent stem cells^48^. Accordingly, stem cell-related mechanisms may also play role in cancer cell dormancy. Indeed, six (CITED2, EPAS1, FOLR1, KDM3A, KLF4 and, SOX9) of the genes in the dormancy interaction network are associated with “stem-cell” term in GO database, and two (EPAS1 and KLF4) are associated with “transcriptional regulation of pluripotent stem-cell” term in REACTOME database.

To provide an independent validation for our findings, we analyzed two additional transcriptomic datasets^49,50^ that were made publicly available after we completed our analyses (Supplementary Tables S1﻿, S2). The analysis of the datasets with the same pipeline led to a combined list of 40 genes (24 genes in one dataset, 21 genes in the other dataset) from our consensus dormancy-interaction network (Supplementary Table S7﻿). Seven of those overlapping genes (ABCG1, CDKN2B, CLU, FBXO32, PLK2, VEGFA, THBS1) are among the top 10 genes reported in Table 1. As another independent validation, we scanned deletion/overexpression phenotypes of the yeast homologs of the genes in the dormancy-interaction network. Homologs of CCNG2, RASAL2, SLC3A2 and ABCG1 genes in the yeast *S. cerevisiae* exhibit haploproficiency, meaning that deletion of one of the alleles provided growth advantage to yeast cells. This is in line with the fact that these genes are all overexpressed in dormancy, implying their possible role in suppressing cell division. Similarly, the overexpression of the yeast homolog of FMNL2 gene from the network leads to lethality, which can show implication of the gene in driving the tumor cells to dormancy state. Therefore, (i) several literature findings for a number of genes in our dormancy-interaction network in terms of their role in dormancy and metastasis, (ii) analysis of two independent transcriptome datasets, (iii) comparison with yeast loss-of-function/overexpression studies provide validations and support the hypothesis that our dormancy–interaction network is a suitable model to explain cancer cell dormancy mechanisms.

Mapping transcriptome data from 21 dormancy–cancer comparisons on three different molecular interaction networks led us to construct a consensus dormancy-interaction network. The dormancy–interaction network introduced in this study provides a molecular framework for dormancy mechanisms by combining information repetitively reported in different cancer–dormancy datasets from literature. A number of the genes captured by the network was previously reported to be linked to dormancy or metastasis, providing a validation for the constructed network. However, majority of the nodes in the network are genes that have not been associated with cancer cell dormancy before. Hence, this network is an important model that will shed light on further studies on extended mechanisms for dormancy. As dormancy increases the resistance to therapy and plays a role in metastasis, targeting the genes featured in this study may increase impact of treatments and prevent recurrence.

## Methods

### Datasets

Transcriptome data of proliferative, dormant or post-dormant cancer cells of human or mouse origin were obtained from the GEO database^51^. The following criteria were taken into consideration in selection of transcriptome data to be included; it should include samples of both dormant and proliferative cancer states, at least two samples should be available from each condition, it should consist of human or mouse samples. A total of 10 datasets were found to meet all the criteria, which led to analysis of 21 dormancy–cancer comparisons (17 for solid tumors, 4 for liquid tumors) since a dataset can include data from multiple cancer cell lines (Supplementary Table ﻿S1). The datasets were first checked for outlier samples by creating PCA graphs for each of the 21 comparisons. Based on PCA results, one control sample from GSE57695 dataset (GSM1386901), one control and one dormant sample from HT55 cell line of GSE114012 dataset (GSM3130646 and GSM3130647) were identified as outliers and removed from the datasets prior to further analysis. Additionally, two datasets published after we completed the analyses in this study were selected as validation datasets and the same procedure was applied to these datasets. To obtain gene expression counts, RNAseq FASTQ files of both datasets were downloaded from The European Nucleotide Archive (ENA) [Project IDs: PRJNA644590 (GSE153944) and PRJNA610898 (GSE146592)]. Adapter sequences were removed from RNAseq fastq files using Trimmomatic (Version 0.39)^52^. Then using STAR (Version 2.7.8a)^53^, the trimmed FASTQ files were mapped to genome. For PRJNA610898 human genome GRCh38 and for PRJNA644590 mouse genome GRCm39 were used^54^. The counts were quantified using featureCounts^55^.

### Statistical analysis of transcriptome data

Differential expression analysis was applied to all 21 dormancy–cancer comparisons from 10 datasets to obtain p-values, adjusted p-values and fold changes of genes. The R package Limma^56^ was used to identify differentially expressed genes in microarray data. Limma analyses were performed using the GEO2R tool of GEO database, which also generates corresponding R scripts. The R package DESeq2^57^ was used for the differential expression analysis of RNAseq data. For the differential expression analysis of single-cell RNAseq data, DESeq2 was combined with R package zinbwave^38^.

### Cellular networks

Human protein–protein interaction (PPI) network was downloaded from BioGRID 3.5.166 (release date: November, 2018)^39^. Duplicate edges and self-loops of interactome were removed by using Cytoscape^40^. Entrez ID of each gene in the interactome was retrieved via bioDBnet^41^, and integrated into the interactome. The final human PPI network consisted of 17,241 edges and 292,471 interactions. Mouse PPI network was obtained from a previous study of our research group^42^, which combined and merged PPI data from different databases. Duplicate edges and self-loops of mouse PPI network were removed, and gene names were converted to Entrez IDs as described above. The final mouse PPI network consists of 7713 nodes and 24,830 interactions.

Gene-regulatory (GR) networks of both human and mouse were downloaded from TRRUST version2^43^ and RegNetwork^44^ databases. The networks from the two databases were merged, and duplicate edges and nodes and self-loops were removed. miRNA interactions, if any, were removed. The final human GR network consists of 6261 nodes, 19,146 edges and 945 transcription factors (TF) while the final mouse GR network has 4010 nodes, 13,485 edges and 1070 transcription factors. A modified TF-free version of GR network, termed TF-free network, was also created and used in the analysis. Here, all genes that were affected by the same TF were represented to be in interaction with each other, and all TF interactions were removed from the network. In this way, the direct effect of the transcription factor is eliminated, but gene regulation information is conserved. Human and mouse TF-free networks consist of 6048 nodes and 2,530,468 edges, and 3767 nodes and 4,622,077 edges, respectively.

### Subnetwork discovery

Subnetwork discovery analysis was performed for all 21 dormancy–cancer comparisons from 10 datasets and 2 validation datasets. Data were mapped on PPI networks, GR networks and TF-free GR networks separately by using KeyPathwayMiner (KPM)^7^ and BioNet^8^ tools.

For KPM analysis, a binarized version of Benjamini–Hochberg corrected p-values was created using the associated p-value cut-off value for each comparison. Binarization was done by assigning the value 1 to significant genes and assigning the value 0 to non-significant genes. The appropriate cut-off values were chosen such that a similar-size subnetwork can be obtained for all comparisons (Supplementary Table S2). The cut-off values of each comparison remained the same across analyses based on protein–protein interactome and gene-regulatory interactome. Thereafter, the stand-alone version of KeyPathwayMiner (KPM 4.0) was executed with the following parameters: Individual Node Exceptions (INEs) strategy, Ant Colony Optimization (ACO) algorithm, *K* = 2 for PPI and K = 8 for GR networks. K is a parameter to define the maximum number of exception (not differentially expressed) nodes allowed in a subnetwork.

BioNet version 1.42.0 was run in R for each comparison. Calculated p-values were used as input, and False Discovery Rate (FDR) cut-off values for each pairwise comparison were selected such that similar-size subnetworks will be produced (Supplementary Table S2). The FDR values of each comparison remained the same across the analyses based on protein–protein and gene-regulatory interactomes.

### Interpretation of subnetworks

In order to interpret the discovered subnetworks, we used three different approaches. Firstly, enrichment analyses were performed by using g:Profiler online tool^45^. g:Profiler allows to perform multiple types of enrichment analyses in one run. It retrieves data from different sources including GeneOntology, KEGG, Reactome and TRANSFAC databases. Its g:GOST module was used to perform functional enrichment analysis on input gene lists with default parameters.

Secondly, genes in all subnetworks were scored based on number of appearances in different subnetworks. To combine mouse and human subnetworks, human orthologues of mouse genes were retrieved via Ensembl/BioMart^46^. In our scoring approach, a gene gets a score of 1 for each subnetwork in which they appeared, and, the final score of a gene is sum of its scores across all constructed subnetworks. Two different scoring approaches were used. In the first scoring approach, called “significance score”, the gene gets a score of 1 if it is identified with at least one of the BioNet or KPM tools in a dormancy–cancer comparison and significantly changed in the comparison. In our second scoring approach, genes are scored for the number of datasets in which they were found. In this scoring, called “dataset score”, a gene gets a score of 1 if it is identified in at least one of all comparisons in a single dataset to minimize bias due to datasets with a higher number of comparisons. A filtering was applied to the scored genes afterwards by using their significance and dataset scores together. Here, genes were filtered if their dataset scores were at least 2 and their significance scores were at least 3. Additionally, we specifically chose genes that are mostly upregulated in dormancy. For this, we required that if the significance score of a gene is 3, it must be upregulated in all of these 3 comparisons. If the score is higher than 3, it must be upregulated in more than half of the subnetworks in which the gene was scored significant. All calculations and rearrangements were performed in R.

Thirdly, Gene Ontology terms in AmiGO database were used to construct lists of mouse and human genes that are associated with autophagy, cell cycle, and extracellular matrix terms^47^. Additionally, a list of dormancy-associated genes in human was manually curated based on abstracts of about 200 articles. Mouse homologs of dormancy genes were identified via Ensembl/BioMart^46^. Then, the genes in the subnetwork score tables were annotated for their association to autophagy, cell cycle, extracellular matrix and dormancy (Supplementary Tables S3﻿, S5). Workflow of computational approach to construct the dormancy-interaction network is shown in Fig. 1.

## Supplementary Information

Supplementary Information.

Supplementary Table 2.

Supplementary Table 3.

Supplementary Table 5.

Supplementary Table 6.

Supplementary Table 7.

## Data Availability

The datasets analysed during the current study are available in the Gene Expression Omnibus (GEO) repository. All data are incorporated into the article and its online Supplementary material.
