# Supplementary material for: Transcriptional landscape of cellular networks reveal interactions driving the dormancy mechanisms in cancer
Source: Sci Rep. 2021 Aug 4;11:15806. doi: 10.1038/s41598-021-94005-x (PMC8339123; doi:10.1038/s41598-021-94005-x)
Supplement: Supplementary file 1 — Supplementary Information. [file 41598_2021_94005_MOESM1_ESM.docx]

**Transcriptional landscape of cellular networks reveal interactions driving the dormancy mechanisms in cancer**

Dilara Uzuner^1^, Yunus Akkoç^2^, Nesibe Peker^2^, Pınar Pir^1^, Devrim Gözüaçık^2,3,4^, Tunahan Çakır^1,*^

^1^ Department of Bioengineering, Gebze Technical University, Kocaeli, 41400, Turkey

^2^ Koç University Research Center for Translational Medicine (KUTTAM), Zeytinburnu 34010, Istanbul, Turkey.

^3^ Koç University School of Medicine, Sariyer 34450, Istanbul, Turkey

^4^ SUNUM Nanotechnology Research and Application Center, Tuzla 34956, Istanbul, Turkey

^*^ Correspondence: tcakir@gtu.edu.tr (T.Ç.)

**Supplemental Information**

**Supplementary Table S1:** Summary of transcriptome datasets.

**Supplementary Table S2:** KPM cut-off and BioNet FDR values of each compared condition and sizes of subnetworks.

**Supplementary Table S3:** Filtered score tables of subnetworks.

**Supplementary Table S4:** Significant scores of Top10 genes in liquid and solid tumor types.

**Supplementary Table S5:** Genes not found in genome-wide PPI network.

**Supplementary Table S6:** Enrichment results of union and intersection of genes that found in filtered score table and interacting in PPI, GR and TF-free GR networks.

**Supplementary Table S7:** Genes found in subnetworks of validation datasets and dormancy-interaction network.

**Supplementary Figure S1:** **For the datasets for which both KPM and BioNet works, the percentage information is given as to which tool identified a gene in the dormancy-cancer interaction network.** Blue bars indicate percent of genes discovered in larger number of subnetworks by BioNet than by KPM, gray bars indicate percent of genes discovered in larger number of subnetworks by KPM than by BioNet. Orange bars indicate the percent of genes discovered in equal number of subnetworks by both tools.

**Supplementary Figure S2: Interacting genes of filtered gene lists.** A) PPI, B) GR, C) TF-free GR genes. Color tones of nodes represent significance score of genes (darker color means higher significance score).

**Supplementary Table S1: Summary of transcriptome datasets**

| **GEO Code** | **Condition** | **Organism** | **Cancer Type** | **Experimental Method** | **Reference** |
| --- | --- | --- | --- | --- | --- |
| **GSE35947** | J82 cell line, dormant vs proliferative | *Homo sapiens* | Bladder Cancer | Microarray | (Hurst et al. 2013) |
|  | JB-V cell line, dormant vs proliferative |  |  |  |  |
| **GSE83142** | RNAseq analysis, dormant vs proliferative | *Homo sapiens* | Acute Lymphoblastic Leukemia | RNAseq | (Ebinger et al. 2016) |
|  | scRNAseq analysis, dormant vs proliferative |  |  | scRNAseq |  |
| **GSE114012** | DLD1 cell line, dormant vs proliferative | *Homo sapiens* | Colorectal Cancer | RNAseq | (Buczacki et al. 2018) |
|  | HCT15 cell line, dormant vs proliferative |  |  |  |  |
|  | HT55 cell line, dormant vs proliferative |  |  |  |  |
|  | RKO cell line, dormant vs proliferative |  |  |  |  |
|  | SW48 cell line, dormant vs proliferative |  |  |  |  |
|  | SW948 cell line, dormant vs proliferative |  |  |  |  |
| **GSE102483** | dormant vs proliferative | *Homo sapiens* | Acute Myeloid Leukemia | Microarray | (Al-Asadi et al. 2017) |
| **GSE20611** | dormant vs post-dormant | *Homo sapiens* | Breast Cancer | Microarray | (Lu et al. 2011) |
| **GSE64262** | dormant vs proliferative | *Homo sapiens* | Prostate Cancer | Microarray | (Ruppender et al. 2015) |
| **GSE77379** | PC3 cell line *in vivo*, dormant vs proliferative | *Homo sapiens* | Prostate Cancer | Microarray | (Shiozawa et al. 2016) |
|  | PC3 cell line *in vitro*, dormant vs proliferative |  |  |  |  |
|  | C42B cell line *in vivo*, dormant vs proliferative |  |  |  |  |
|  | C42B cell line *in vitro*, dormant vs proliferative |  |  |  |  |
| **GSE57695** | dormant vs proliferative | *Mus musculus* | Myeloma | Microarray | (Lawson et al. 2015) |
| **GSE112094** | dormant vs proliferative | *Mus musculus* | Breast Cancer | RNAseq | (Vera-Ramirez et al. 2018) |
| **GSE131890** | D2.OR (3D) vs D2.A1 (3D) cell line, dormant vs proliferative | *Mus musculus* | Breast Cancer | Microarray | (La Belle Flynn et al. 2019) |
|  | D2.OR (3D) vs D2.OR (2D) cell line, dormant vs proliferative |  |  |  |  |
| **GSE146592*** | dormant vs proliferative | *Homo sapiens* | Acute Myeloid Leukemia | RNAseq | (Duy et al. 2021) |
| **GSE153944*** | dormant vs proliferative | *Mus musculus* | Non-small cell lung cancer | RNAseq | (Perego et al. 2020) |

*Validation dataset

**Supplementary Table S4: Significant scores of Top10 genes in liquid and solid tumor types.**

| **Genes of top 10 list**  **(Table 1)** | **Solid cancer score (PPI / GRN / TF-free GRN)** | **liquid cancer score (PPI / GRN / TF-free GRN)** |
| --- | --- | --- |
| **CLU** | 8/6/7 | 1/1/1 |
| **CDKN2B** | 4/5/5 | 1/1/1 |
| **HIST2H2BE** | 5/5/5 | 1/1/1 |
| **FBXO32** | 6/6/6 | 0/0/0 |
| **CTSB** | 5/6/6 | 0/0/0 |
| **THBS1** | 5/5/5 | 0/0/0 |
| **VEGFA** | 4/5/5 | 0/0/0 |
| **PLK2** | 4/4/4 | 1/1/1 |
| **BMF** | 0/4/4 | 0/1/1 |
| **EPAS1** | 0/5/5 | 0/0/0 |
| **HIST1H1C** | 5/0/0 | 1/0/0 |
| **APP** | 5/0/0 | 2/0/0 |
| **OPTN** | 6/0/4 | 0/0/2 |
| **PLAUR** | 4/4/4 | 1/1/1 |
| **NEU1** | 0/0/7 | 0/0/0 |
| **HSPA1B** | 0/0/5 | 0/0/1 |
| **NR1D1** | 4/1/5 | 0/0/1 |
| **ABCG1** | 3/4/5 | 1/1/1 |


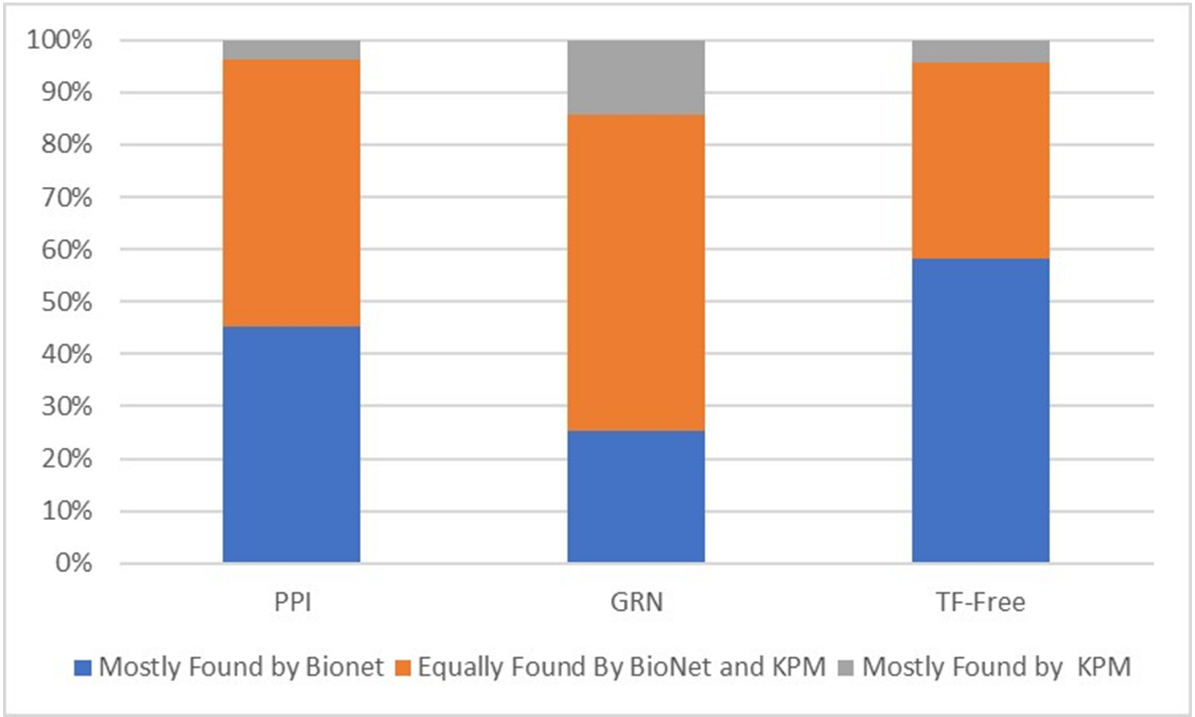


**Supplementary Figure S1:** **For the datasets for which both KPM and BioNet works, the percentage information is given as to which tool identified a gene in the dormancy-cancer interaction network.** Blue bars indicate percent of genes discovered in larger number of subnetworks by BioNet than by KPM, gray bars indicate percent of genes discovered in larger number of subnetworks by KPM than by BioNet. Orange bars indicate the percent of genes discovered in equal number of subnetworks by both tools.


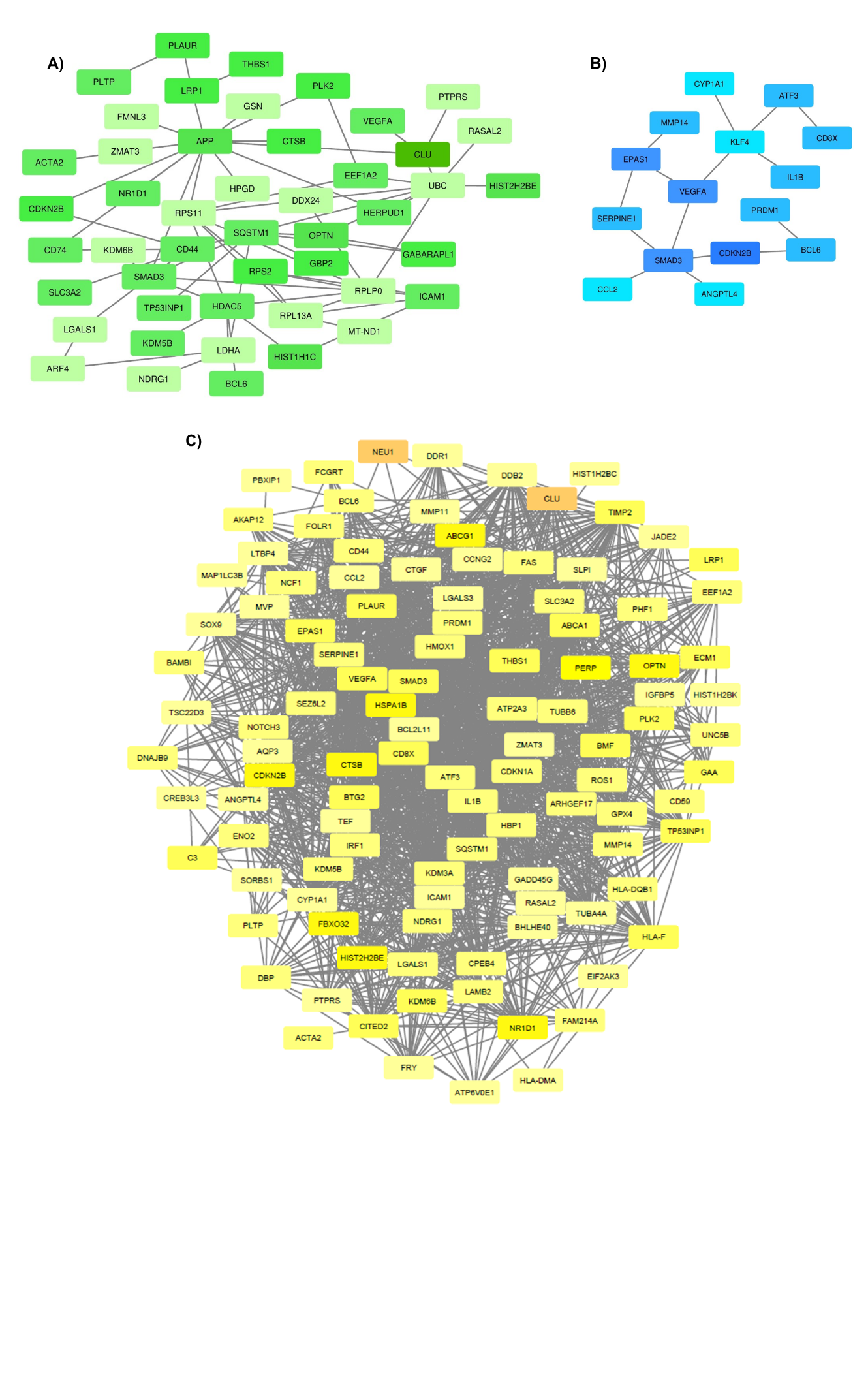


**Supplementary Figure S2:** Interacting genes of filtered gene lists. A) PPI, B) GR, C) TF-free GR genes. Color tones of nodes represent significance score of genes (darker color means higher significance score).

**References**

Al-Asadi, Mazin Gh, et al. (2017), 'A molecular signature of dormancy in CD34(+)CD38(-) acute myeloid leukaemia cells', *Oncotarget,* 8 (67), 111405-18.

Buczacki, S. J. A., et al. (2018), 'Itraconazole targets cell cycle heterogeneity in colorectal cancer', *J Exp Med,* 215 (7), 1891-912.

Ebinger, S., et al. (2016), 'Characterization of Rare, Dormant, and Therapy-Resistant Cells in Acute Lymphoblastic Leukemia', *Cancer Cell,* 30 (6), 849-62.

Hurst, R. E., et al. (2013), 'Suppression and activation of the malignant phenotype by extracellular matrix in xenograft models of bladder cancer: a model for tumor cell "dormancy"', *PLoS One,* 8 (5), e64181.

La Belle Flynn, Alyssa, et al. (2019), 'Autophagy inhibition elicits emergence from metastatic dormancy by inducing and stabilizing Pfkfb3 expression', *Nature communications,* 10 (1), 3668-68.

Lawson, M. A., et al. (2015), 'Osteoclasts control reactivation of dormant myeloma cells by remodelling the endosteal niche', *Nat Commun,* 6, 8983.

Lu, X., et al. (2011), 'VCAM-1 promotes osteolytic expansion of indolent bone micrometastasis of breast cancer by engaging alpha4beta1-positive osteoclast progenitors', *Cancer Cell,* 20 (6), 701-14.

Ruppender, N., et al. (2015), 'Cellular Adhesion Promotes Prostate Cancer Cells Escape from Dormancy', *PLoS One,* 10 (6), e0130565.

Shiozawa, Yusuke, et al. (2016), 'The marrow niche controls the cancer stem cell phenotype of disseminated prostate cancer', *Oncotarget,* 7 (27), 41217-32.

Vera-Ramirez, L., et al. (2018), 'Autophagy promotes the survival of dormant breast cancer cells and metastatic tumour recurrence', *Nat Commun,* 9 (1), 1944.

Duy, C., et al. (2021), 'Chemotherapy Induces Senescence-Like Resilient Cells Capable of Initiating AML Recurrence', *Cancer Discov,* 11 (6), 1542-61.

Perego, M., et al. (2020), 'Reactivation of dormant tumor cells by modified lipids derived from stress-activated neutrophils', *Sci Transl Med,* 12 (572).
